# Associations between tertiary lymphoid structure density and immune checkpoint inhibitor efficacy in solid tumors: systematic review and meta-analysis

**DOI:** 10.3389/fimmu.2024.1414884

**Published:** 2024-10-31

**Authors:** Bin Jiang, Zhuo Wu, Yang Zhang, Xueying Yang

**Affiliations:** Department of Thoracic Surgery, The Fourth Affiliated Hospital of China Medical University, Shenyang, China

**Keywords:** tertiary lymphoid structures (TLS), immune checkpoint inhibitors, immunotherapy, solid tumors, PD1/PDL1

## Abstract

**Background:**

Tertiary lymphoid structures (TLS) play a crucial role in tumor immunity, yet their relationship with the efficacy of immune checkpoint inhibitors (ICI) in cancer therapy is not fully understood. This study aims to systematically evaluate how TLS density influences treatment outcomes in cancer patients receiving ICI therapy.

**Methods:**

The PubMed, Embase, Cochrane Library, and Web of Science databases were searched for eligible studies published before January 22, 2024. Our analysis encompassed odds ratios (ORs) for response rates (RRs) and hazard ratios (HRs) for progression-free survival (PFS), each with their respective 95% confidence intervals (CIs).

**Results:**

Our meta-analysis, including 19 clinical trials with 1,752 patients, identified a strong correlation between high TLS density and increased RR to ICIs (OR= 2.99, 95% CI: 2.14-4.18, *P* < 0.001). Furthermore, a higher TLS density was associated with prolonged PFS (HR=0.75, 95% CI: 0.63-0.88, *P* < 0.001). Specifically, in the context of non-small cell lung cancer (NSCLC), breast cancer (BC), renal cell carcinoma (RCC), esophageal cancer (EC), and urothelial carcinoma (UC), a significant relationship was observed between high TLS density and better ICI efficacy. Publication bias did not affect the integrity of our conclusions. Sensitivity analysis further reinforced the reliability of our aggregated outcomes.

**Conclusion:**

Our meta-analysis underscores the predictive role of TLS density in determining the RR and PFS among cancer patients undergoing ICI therapy. These results highlight the prognostic significance of TLS, suggesting its potential as a biomarker for guiding treatment decisions, even in tumor types traditionally considered ICI-resistant. Clinicians are recommended to assess TLS density as a part of patient evaluation to optimize ICI therapy initiation.

**Systematic review registration:**

https://www.crd.york.ac.uk/prospero/, identifier CRD42023439875.

## Introduction

Since their introduction, immune checkpoint inhibitors (ICIs) have emerged as a critical treatment for cancer, supplementing traditional approaches such as surgery, chemotherapy, and radiation therapy. However, the efficacy of ICIs remains limited, with response rates for most tumors lying between 10% and 40% (1). This disparity underscores the need for reliable biomarkers to predict ICI therapy outcomes, making precision in immunotherapy a critical area of clinical research. Initially, PD-1/PD-L1 expression was explored for this purpose, yet predictive accuracy has been suboptimal (2). Tertiary lymphoid structures (TLS)—comprising lymphocyte and myeloid cell aggregations within inflamed tissues, akin to secondary lymphoid organs and frequently observed in tumor proximity— have shown promise. Notably, the presence of TLS in pre-treatment biopsy samples has been linked with increased ICI responses across various cancers (3–6). For instance, in the study by Lucile Vanherske, patients with mature TLS, regardless of PD-L1 expression, exhibited a 40.3% response rate to ICI therapy (7). Despite these indications of TLS as a potential ICI efficacy predictor, substantial epidemiological evidence remains scarce. Our meta-analysis aims to demonstrate that TLS density can effectively predict the response and therapeutic effect of ICI.

## Methods

### Protocol and registration

This systematic review and meta-analysis adhered to the PRISMA guidelines (http://www.prisma-statement.org/) and was registered on PROSPERO (CRD42023439875) (**Supplementary Table S1**).

### Data sources and search strategy

We conducted our search across four primary databases: PubMed, Embase, Cochrane Library, and Web of Science, supplemented by additional internet searches to locate further relevant studies. The search was last updated on January 22, 2024. The following search terms were used: (**Supplementary Table S2**).

### Selection criteria

Studies were selected based on the following criteria:

#### Participants

Patients with advanced malignant tumors (any solid tumor) undergoing ICI therapy were included.

#### Intervention

We included studies that assessed the use of ICIs, either as monotherapy or in combination with other anticancer agents, irrespective of the administration route.

#### Outcomes

We included studies that evaluated potential correlations between TLS and treatment outcomes in cases where ICIs were part of the regimen.

#### Study design

We included observational cohort and case−control studies, excluded reviews, meta-analyses, case reports, or guidelines. We excluded studies in which a 2 × 2 table between TLS density and the outcome of ICI treatment could not be constructed. We also excluded studies with insufficient data or no relevant information provided.

Two independent reviewers screened the titles and abstracts, and the full texts of the studies that met the inclusion criteria were obtained. Any disagreements encountered during the screening process were resolved through discussion and, if necessary, with the assistance of a third reviewer.

### Data extraction

The extracted variables, when available, included: Name of the first author, Year of publication, Study sample size, Ratio of patients with high/low TLS, Number of responding patients, Type of cancer, TLS determination method, Cut-off criteria for TLS, Cut-off value, Stage of tumor, ICI application, ICI therapy, Response rate (RR) and survival analysis for TLS status subgroups, including hazard ratios (HRs) and 95% CIs for progression-free survival (PFS). In cases where HRs and 95% CI were not provided, Engauge Digitizer software version 4.1 was employed to analyze Kaplan– Meier curves, facilitating the extraction of multiple survival rates to estimate HRs and 95% CIs. Data extraction was independently conducted by two researchers, with any discrepancies resolved through discussion with a third researcher.

We performed comparative analyses between high and low TLS groups based on historical data. We classified TLS-high and TLS-low subgroups using three distinct cutoff criteria (presence, density, and signature score). The included studies provided specific TLS detection method, such as hematoxylin and eosin (H&E) staining, immunohistochemistry (IHC), etc.

For classifications based on presence and density, two detection methods for TLS were employed: H&E staining, and IHC. In H&E staining, TLS is generally defined as dense lymphocyte aggregation, while in IHC, CD20-enriched areas occasionally accompanied by CD3, CD21, or CD8 were defined as TLS. We ensured that TLS at all stages of maturity (both mature and immature) were included in each study.

We evaluated the impact of TLS on the response to ICI therapy by assessing the patient RR. Complete response, partial response, and objective response were classified as responses, Objective response included both complete response and partial response. Complete response was defined as complete disappearance of all measurable disease, partial response as a reduction in tumor size comparable to that defined by Response Evaluation Criteria in Solid Tumors (RECIST) criteria.

Stable disease, absence of progression, and progressive disease were considered non-responses. Progressive disease was either clinical progression or tumor growth, stable disease or absence of progression was patients who did not meet RECIST criteria for either disease progression or objective response.

The overall proportion of responses was defined as the RR. In studies where only the objective response rate (ORR) was calculated, ORR was considered equivalent to RR.

### Statistical analysis

We calculated ORs using random-effects Mantel-Haenszel meta-analysis models, based on the TLS content in patients’ biopsy specimens. PFS outcomes were reported as HRs with 95% CIs.

Statistical analyses were conducted using Review Manager 5.4 (Cochrane Collaboration, Oxford, UK) and STATA 17.0 (StataCorp LP, College Station, Texas). To evaluate heterogeneity across all meta-analyses, the Cochrane Q*P* -value and *I*² statistic were employed. Significant heterogeneity was indicated by a *P*-value < 0.05 or *I*² > 50%, prompting the use of a random-effect model to integrate the results. In the absence of significant heterogeneity, a fixed-effect model was applied. Statistical significance was set at a *P*-value < 0.05. Publication bias was examined through Egger’s test, and the trim and fill method was applied to amend the results in the presence of significant publication bias.

### Quality evaluation

The quality of included studies was evaluated using the Newcastle-Ottawa Scale, while the level of evidence was assessed according to the standards set by the Oxford Centre for Evidence-Based Medicine. Each study received independent ratings from two researchers, with scores up to a maximum of 9, focusing on patient selection, outcome assessment, and comparability. This independent evaluation by two researchers aimed to minimize bias. In cases of scoring discrepancies, the study was re-evaluated and a consensus reached through discussion among the authors.

## Results

### Search results

**Figure 1:**
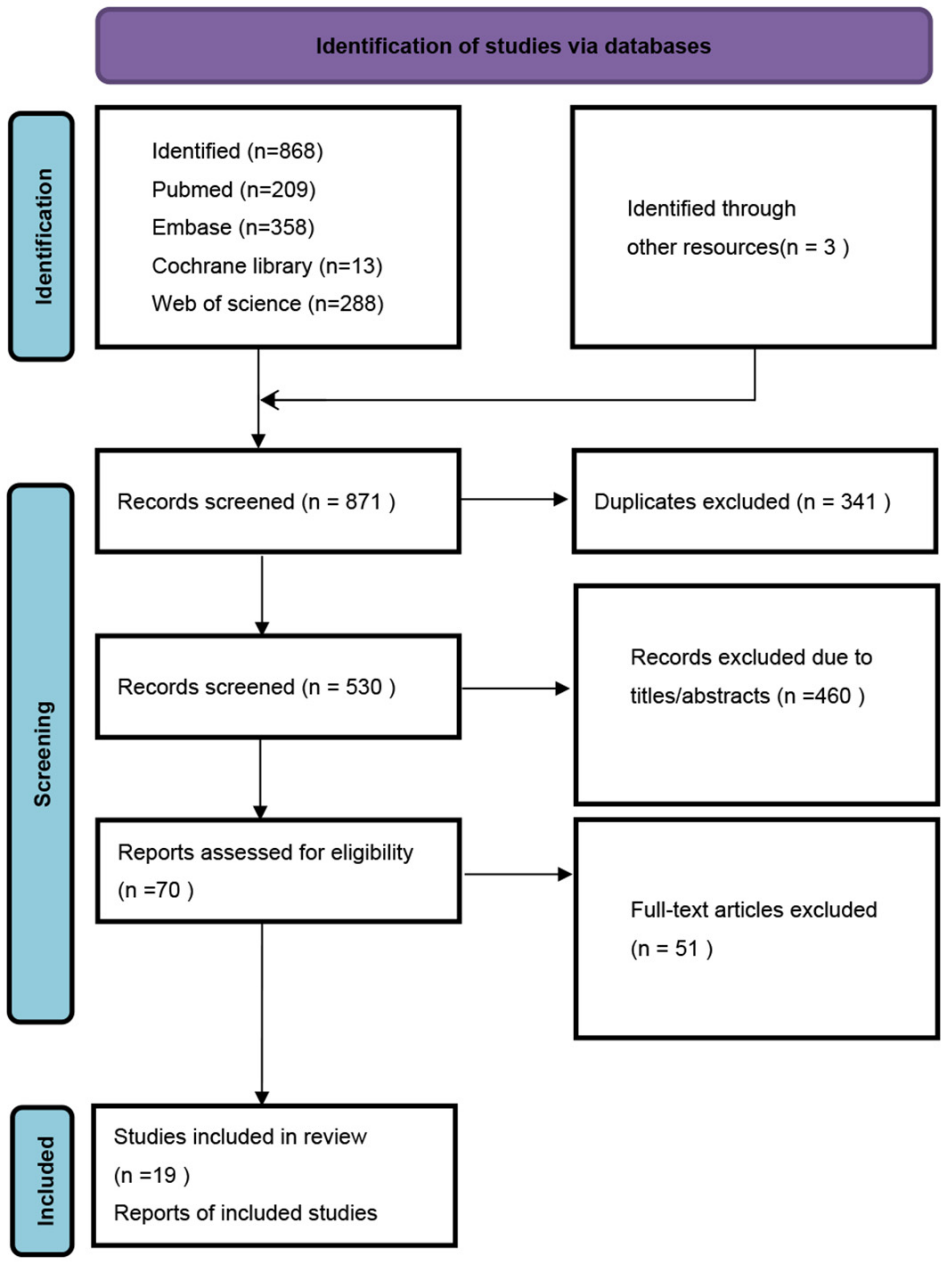
The flow diagram of identifying eligible studies.

A total of 871 studies were identified through electronic searches. From these, 341 duplicates were removed, along with 460 studies deemed irrelevant based on their titles and abstracts. After a detailed review of the full texts, 51 studies were further excluded. Consequently, 19 studies encompassing 1,752 patients with solid tumors who received ICI treatment were selected for inclusion in our meta-analysis. The entire selection process is illustrated in the provided PRISMA flowchart (**Figure 1**).

### Study characteristics and quality assessment

The studies included in this meta-analysis are detailed in (**Table 1**). The compilation features five studies on non-small cell lung cancer (NSCLC), three on urothelial carcinoma (UC), two each dedicated to gastric cancer (GC) and renal cell carcinoma (RCC), and one study each on breast cancer (BC), esophageal carcinoma (EC), thoracic tumors, hepatocellular carcinoma (HCC), nasopharyngeal carcinoma (NPC), and melanoma. TLS-high and TLS-low subgroups using three distinct cutoff criteria (presence, density and signature score) as enumerated in (**Table 1**).

**Table 1:** Characteristics of 1752 patients with solid tumors treated with ICI.

Study	Region	Sample size	TLS-high/low	Response number	Cancer types	Determination method of TLS	Cut-off criteria	Cut-off value	NOS score s	Outcome measures	Stage of tumor	ICI application	ICI therapy
Jianjun Gao.2020 (8)	USA	26	13/13	13	UC	IHC	Density	0.155 TLS mm−2	6	RR, PFS	N. A	Neoadjuvant therapy	Anti-PD-L1 plus anti-CTLA-4
Lucile Vanherseck. 2021 (7)	France	328	105/223	78	Malignant tumor	H&E+IHC	Presence	0	8	RR	Stage IV	Systemic therapy	Anti-PD1/PD-L1 monoclonal antibodies
Xiaoyan Sun.2022 (9)	China	40	34/6	18	NSCLC	H&E+IHC	Density	TLS score	6	RR	Stage II-IIIA	Neoadjuvant therapy	Anti-PD-1 antibody plus chemotherapy
Takuya Mori.2022 (10)	Japan	19	9/10	3	GC	IHC	Density	Median percentage area (1.24%)	8	RR, PFS	Stage I-IV	Adjuvant therapy	Nivolumab
Jieqiong Liu. 2022 (11)	China	34	14/20	15	BC	IHC	Density	Mean area ≥30,000μm2	6	RR	Unresectable recurrent or metastatic	Systemic therapy	Camrelizumab combined with Apatinib and Eribulin
Y. Hayashi. 2023 (12)	Japan	34	17/17	9	EC	H&E+IHC	Density	0.325/mm2	8	RR, PFS	Stage I-IV	Adjuvant therapy	Anti-PD-1 antibody monotherapy
T. R. Cottrell. 2018 (13)	USA	20	11/9	16	NSCLC	H&E	Presence	0	6	RR	Stage I–IIIA	Neoadjuvant therapy	Nivolumab (anti- PD-1)
Justine Gantzer.2022 (14)	France	4	1/3	1	Thoracic tumors	IHC	Presence	0	6	RR	N. A	Systemic therapy	Immune checkpoint inhibitor
Shu D.H. 2022 (15)	USA	9	5/4	4	HCC	12-chemokine gene signature	Signature score	Hierarchic al clustering	6	RR	Locally advanced	Neoadjuvant therapy	Nivolumab and cabozantinib
Xingchen Li. 2022 (16)	USA	298	78/220	69	UC	12-chemokine gene signature	Signature score	Top 25%	6	RR	Locally advanced or metastatic	Systemic therapy	Atezolizumab administration
Lucia Carril Ajuria.2022 (17)	France	274	126/148	66	RCC	IHC	Density	2 TLS	6	RR, PFS	Metastatic	Systemic therapy	Nivolumab monotherapy
Li Yuan.2023 (18)	China	21	10/11	13	NPC	H&E+IHC	Density	Mean area	6	RR	Recurrent/metastatic	Systemic therapy	Immunotherapy combined with antiangiogenic targeted therapy
Kazumasa Komura. 2023 (5)	Japan	97	23/74	22	UC	IHC	Presence	0	6	RR, PFS	Metastatic	Systemic therapy	Checkpoint inhibitors Pembrolizumab
Nicole L. Edmonds. 2022 (3)	USA	19	1/18	2	Melanoma	IHC	Presence	0	6	RR	Advanced melanoma	Systemic therapy	PD-1 blockade Pembrolizumab
Karlijn Hummelink. 2022 (19)	Netherland s	91	30/61	20	NSCLC	IHC	Presence	0	6	RR	Stage IV	Systemic therapy	PD-1 blockade Monotherapy
Takuya Mori.2021 (20)	Japan	10	3/7	2	GC	IHC	Density	CD20+ B cells (1.59 per field)	8	RR	Stage II-IV	Adjuvant therapy	PD-1 blockade with nivolumab
Wenhao Xu.2023 (21)	China	230	65/165	39	RCC	H&E+IHC	Presence	0	6	RR, PFS	Metastatic	Adjuvant therapy	Tyrosine kinase inhibitors (TKIs) and ICIs combination therapy
Fuhao Xu.2023 (4)	China	106	93/13	64	NSCLC	H&E+IHC	Presence	0	6	RR	Stage IB to IIIB	Neoadjuvant therapy	PD-1 inhibitors combined with taxanes and platinum-based drugs
Ying Liu.2023 (6)	China	80	37/43	30	NSCLC	H&E	Density	5 TLSs	6	RR, PFS	Stage IB- IIIB	Neoadjuvant therapy	Immune checkpoint inhibitors plus platinum-based chemotherapy

NSCLC, non-small cell lung cancer; UC, urothelial carcinoma; GC, gastric cancer; RCC, renal cell carcinoma; BC, breast cancer; EC, esophageal carcinoma; thoracic tumors, HCC, hepatocellular carcinoma; NPC, nasopharyngeal carcinoma; hematoxylin/eosin (H&E) staining, IHC, immunohistochemistry; RR, response rate; PFS, progression-free survival; N.A, not available.

All studies provide RR data, while only seven studies provide PFS data. They are all cohort studies, with quality ratings between six to eight stars out of nine, with none omitted from the meta-analysis.

### Effect of TLS-high on RR

**Figure 2:**
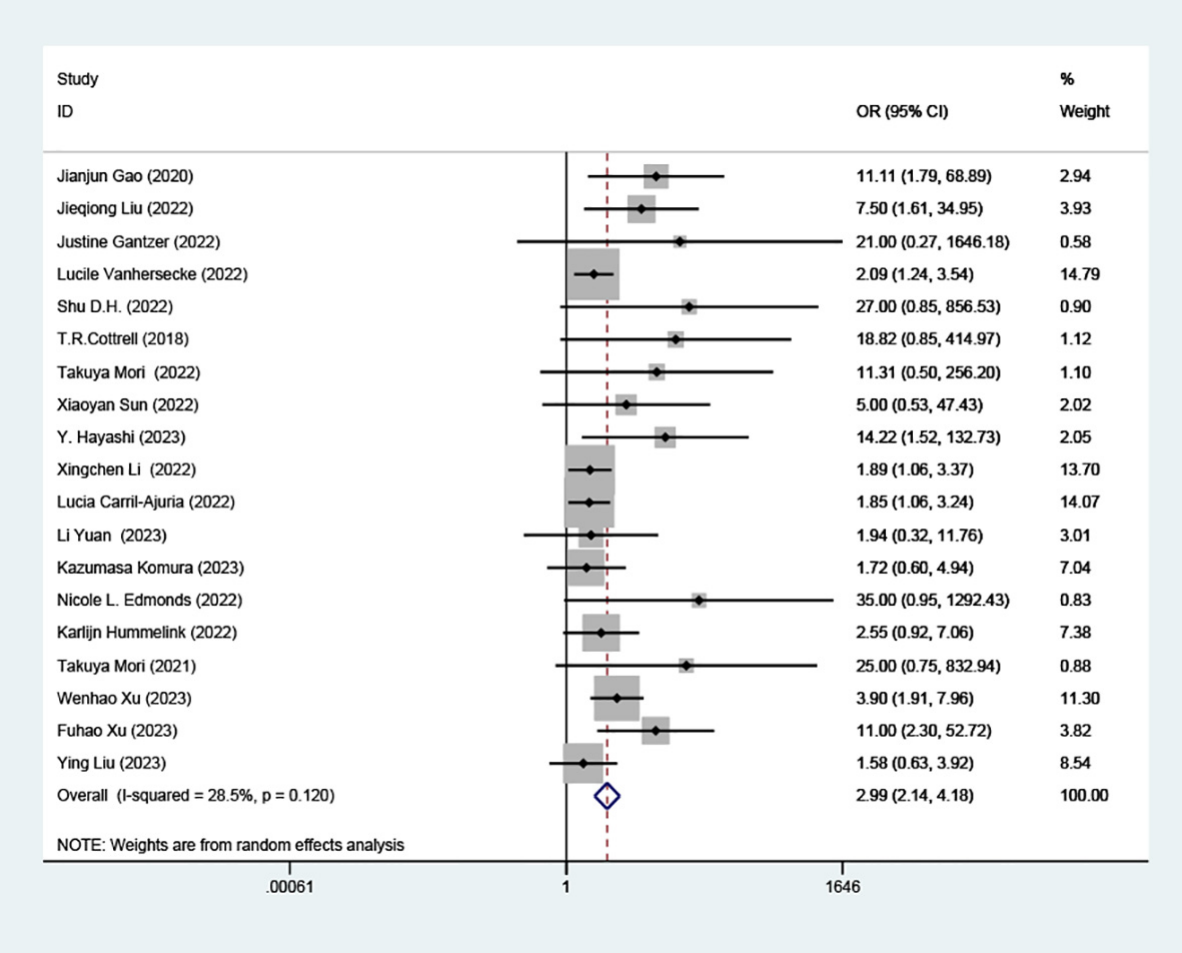
Comparison of response rates between TLS-high and TLS-low patients treated with ICI. OR, odd ratio; CI, confidence interval.

Our analysis revealed a statistically significant correlation between high TLS and increased RR in patients treated with ICIs (OR=2.99; 95% CI: 2.14-4.18, *P* < 0.001), as depicted in (**Figure 2**).

### Effect of TLS-high on PFS

**Figure 3:**
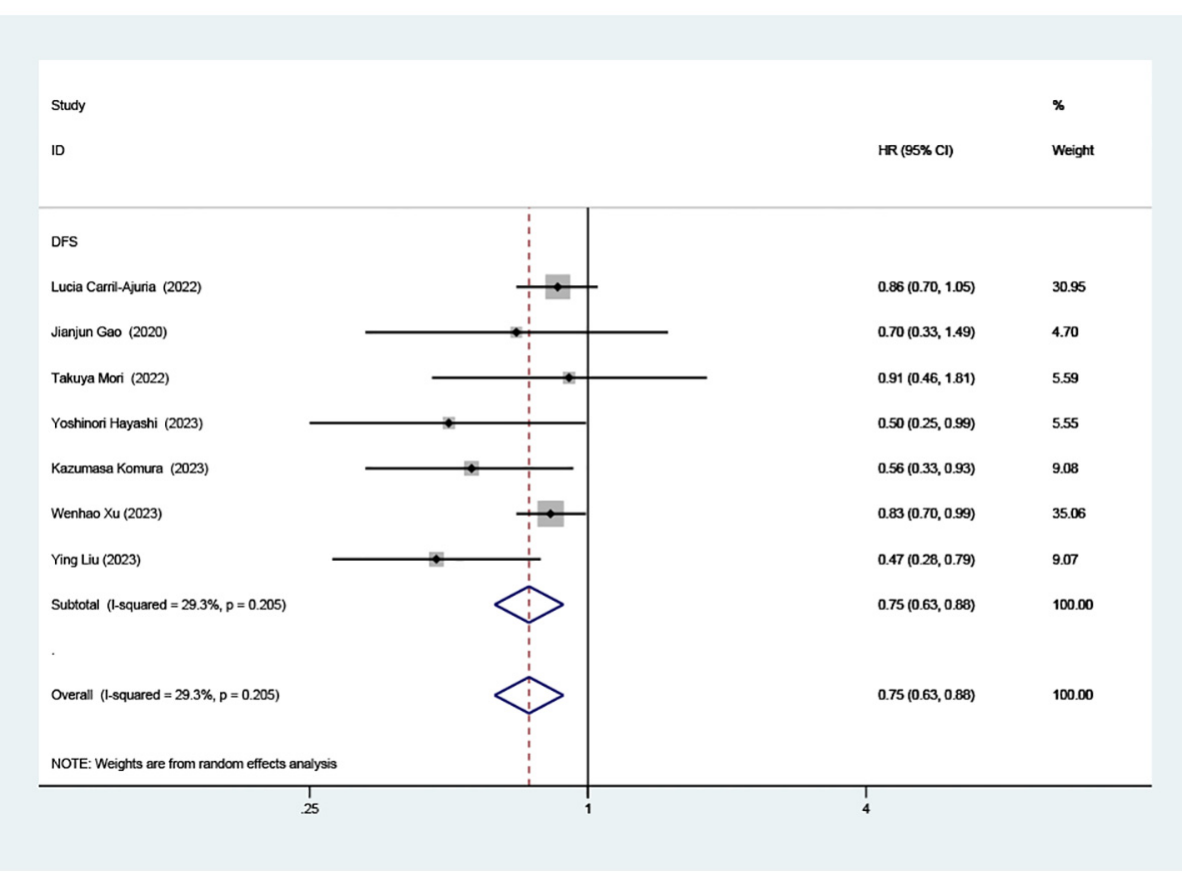
Estimates of progressive-free survival in patients treated with ICI and high TLS. HR, hazard ratios; CI, confidence interval.

Data from seven cohorts, covering 769 patients, provided insights into PFS. Our analysis highlighted a significant and positive relationship between elevated TLS density and extended PFS in patients undergoing ICI therapy (HR=0.75, 95% CI: 0.63-0.88, *P* < 0.001), as illustrated in (**Figure 3**).

### Effect of high TLS on RR according to presence, density and signature score

**Figure 4:**
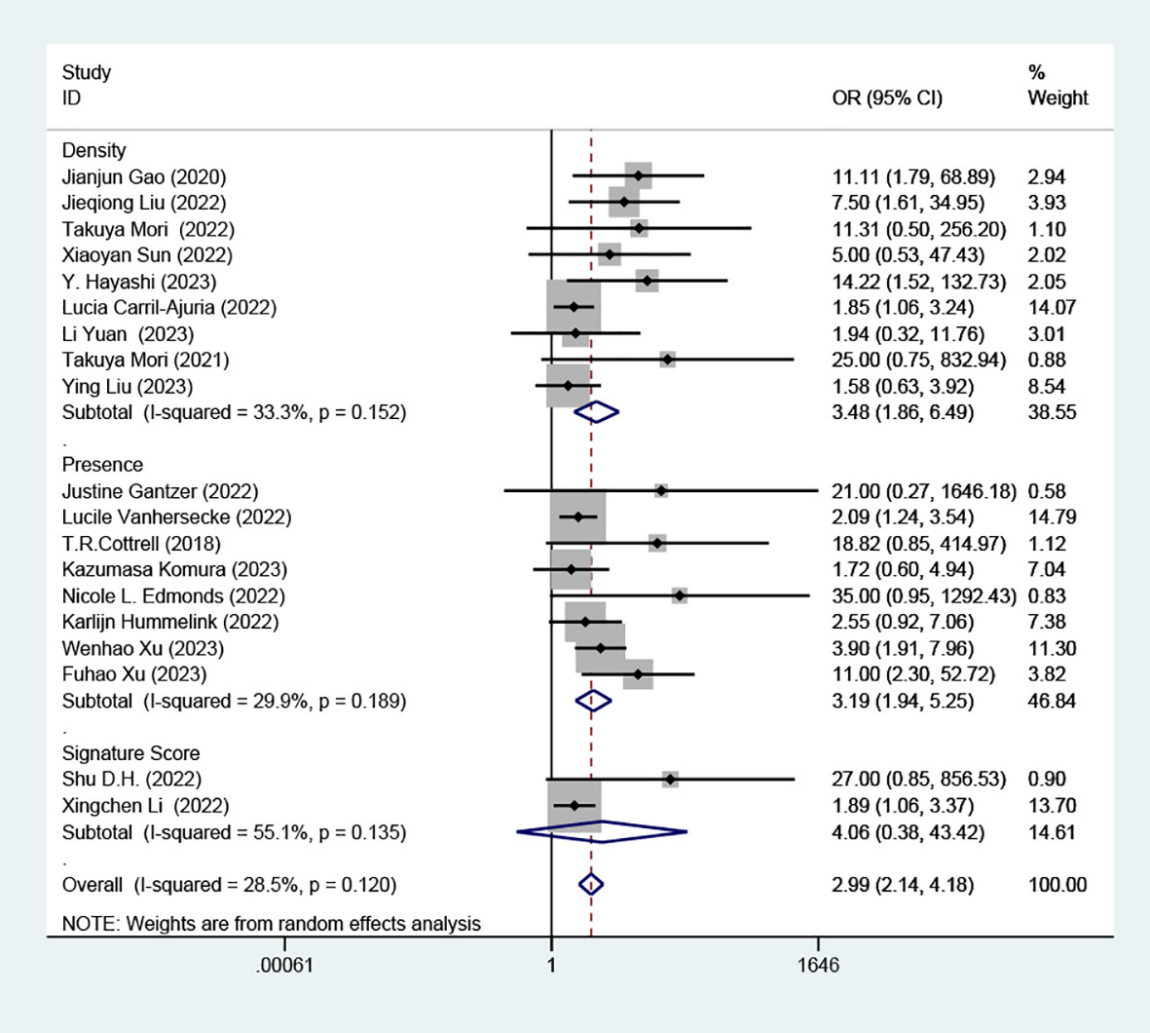
Comparison of response rates between TLS-high and TLS-low patients treated with ICI according to presence, density and signature score.

Subgroup analyses revealed a distinct association between elevated TLS levels and increased RR, categorized by presence (OR=3.19; 95% CI: 1.94-5.25, *P* < 0.001) and density (OR=3.48; 95% CI: 1.86-6.49, *P* < 0.001). However, these analyses found no significant association in the subgroup defined by signature score, (OR =4.06, 95% CI: 0.38-43.42, *P*=0.247), as depicted in (**Figure 4**).

### Effect of high TLS on RR according to tumor classification

**Figure 5:**
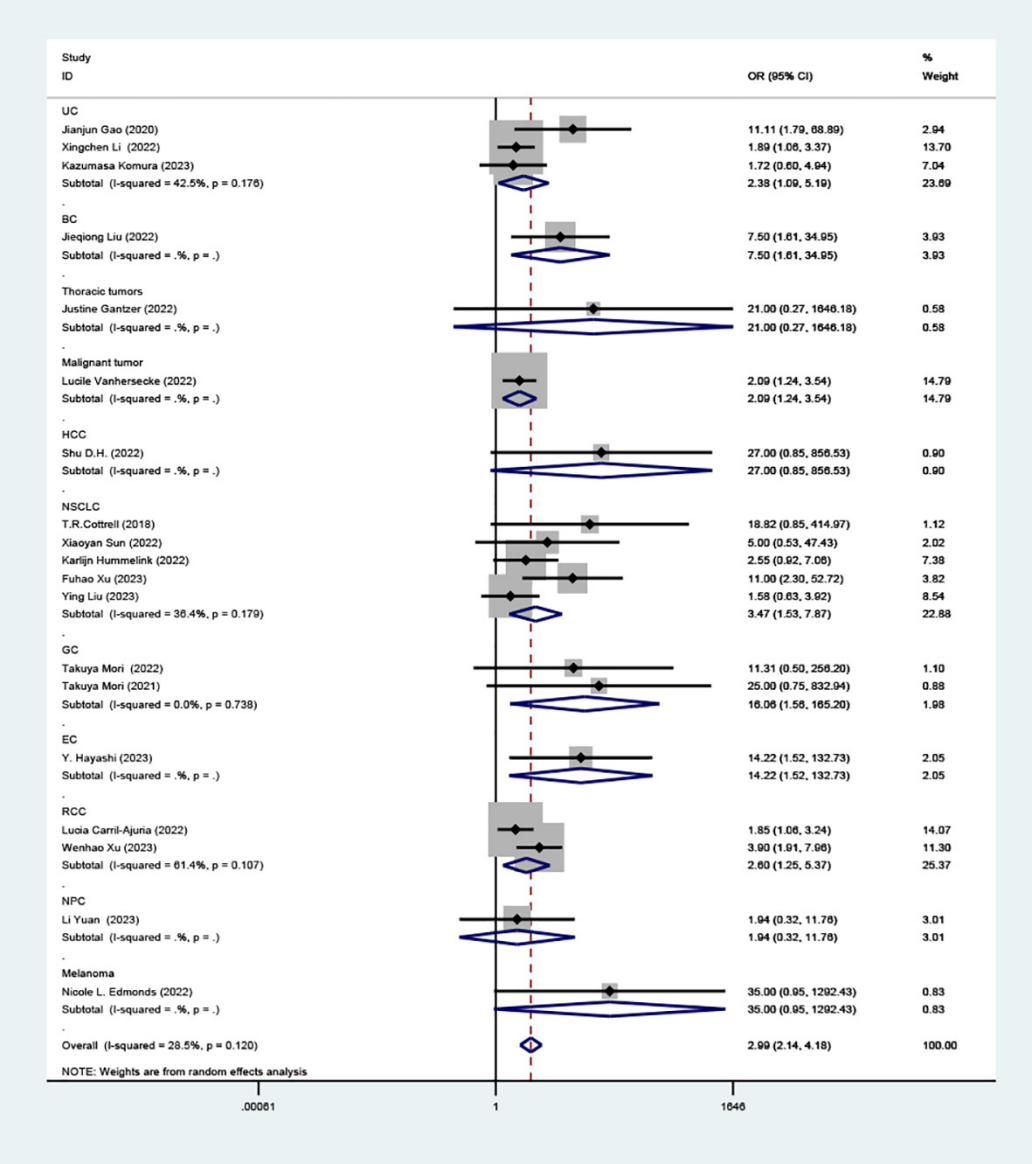
Comparison of response rates between TLS-high and TLS-low patients treated with ICI as subgroups according to tumor classification.

Our detailed analysis focused on specific cancers showed: UC (OR=2.38, 95% CI: 1.09-5.19, *P*=0.029), BC (OR= 7.50, 95% CI: 1.61-34.95, *P*=0.01), HCC (OR=27.00 95% CI: 0.85-856.53, *P*=0.062), NSCLC (OR=3.47, 95% CI: 1.53-7.87, *P*=0.003), GC (OR=16.06, 95% CI: 1.56-165.20, *P*=0.020), EC (OR=14.22, 95% CI: 1.52-132.73, *P*=0.02), RCC (OR=2.60, 95% CI: 1.25-5.37, *P*=0.01), NPC (OR=1.94, 95% CI: 0.32- 11.76, *P*=0.469), melanoma (OR=35.00, 95% CI: 0.95-1292.43, *P*=0.053) as shown in (**Figure 5**). This indicates that TLS density is a strong predictor of ICI response in NSCLC. However, there wasn’t a significant association between TLS density and response rates in HCC, NPC, and melanoma.

Subgroup analyses based on ICI application settings (adjuvant therapy, neoadjuvant therapy, systemic therapy) and ICI therapy approaches (ICI monotherapy, ICI combined with chemotherapy, ICI combined with other anticancer drugs) revealed no significant differences between subgroups, with low heterogeneity. (**Supplementary Figures S1**, **S2**).

### Effect of high TLS on PFS according to tumor classification

**Figure 6:**
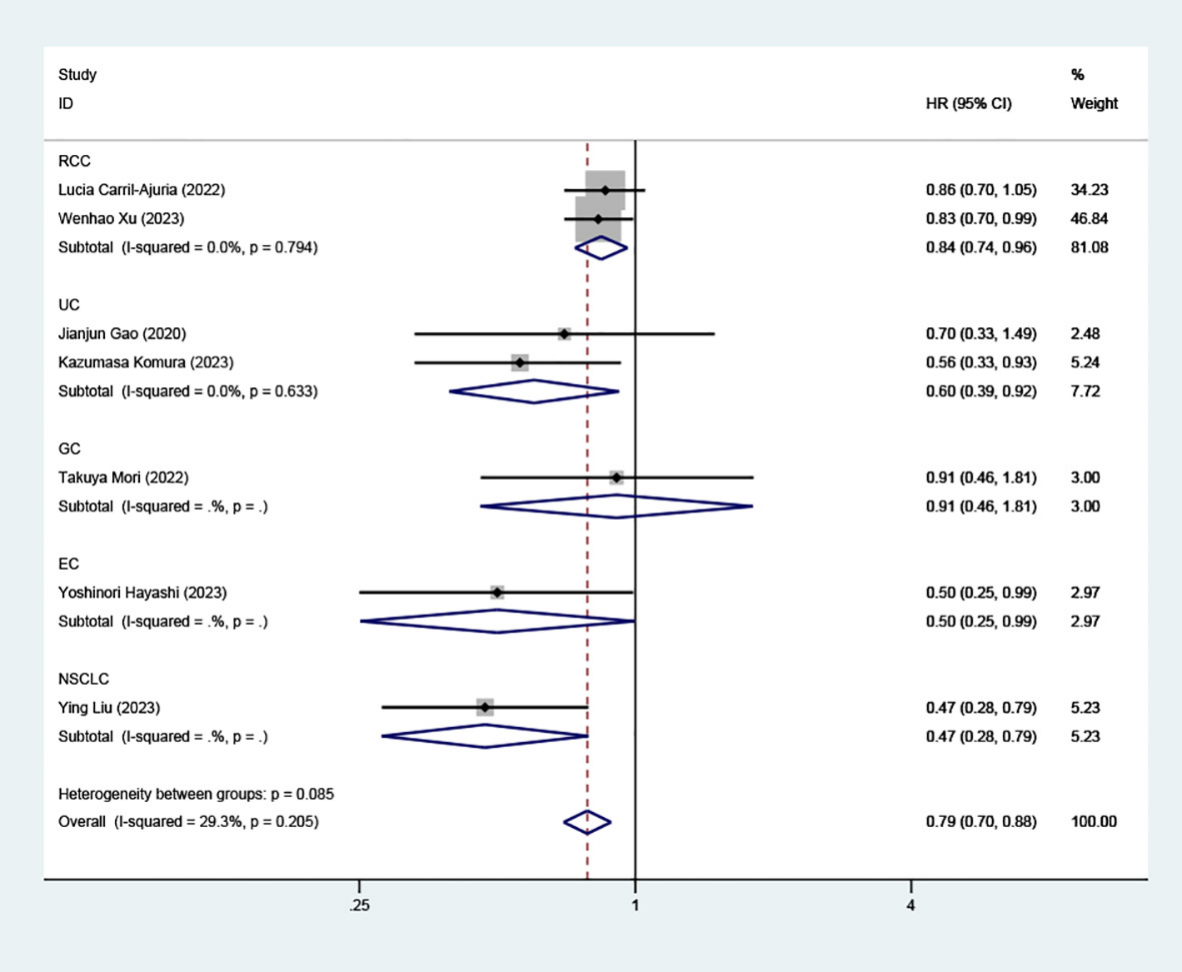
Comparison of progressive-free survival between TLS-high and TLS-low patients treated with ICI as subgroups according to tumor classification.

Subgroup analysis on the association between high TLS density and PFS showed significant findings in RCC (HR=0.84, 95% CI: 0.74-0.96, *P*=0.011), UC (HR=0.60, 95% CI: 0.39-0.92, *P*=0.020), and EC (HR=0.50, 95% CI: 0.25-0.99, *P*=0.048), as well as NSCLC (HR=0.47, 95% CI: 0.28-0.79, *P*=0.004), as illustrated in (**Figure 6**). However, in GC patients, the link between high TLS density and prolonged PFS did not reach statistical significance (HR=0.91, 95% CI: 0.46- 1.81, *P*=0.787).

### Publication bias

To assess the potential for publication bias, we constructed a funnel plot correlating each trial’s effect size with its standard error (see appendix). Funnel plot asymmetry was evaluated using Egger’s tests, with significant publication bias defined by a *P*-value < 0.1. We employed the trim-and-fill method to estimate the influence of publication bias on the interpretation of our findings. The studies exhibited mild heterogeneity, indicated by an *I*² value of 28.5% for the OR. (**Supplementary Figure S3**).

In addition, in 7 articles providing PFS, Egger tests indicated no significant publication bias. The analysis suggested minimal heterogeneity among the studies, with an *I*² value of 29.3% for the HR.

In the various subgroup analyses, the study’s stability remained unaffected by differences in cut-off criteria (*I²*=28.5%, *P*=0.120), tumor classification (*I²*=29.5%, *P*=0.157), ICI application (*I²*=28.5%, *P*=0.120) or ICI therapy (*I²*=30.8%, *P*=0.137), thereby ensuring the reliability of our findings.

## Discussion

Our study establishes a strong connection between TLS density and the efficacy of ICI therapy, highlighting the significant role of TLS density as a predictive marker for ICI response. Through rigorous analysis, our findings have been consistently validated across different cut-off criteria and a diverse array of cancer types, particularly demonstrating solid applicability in UC and RCC. The methods used to classify TLS— specifically presence and density—were found to strongly support our conclusions, underscoring their relevance in predicting therapy outcomes. This research positions TLS density as an indispensable tool for clinicians in devising personalized immunotherapy strategies, aiming to predict both the response rates and prognoses of patients.

TLS is an important component of the tumor microenvironment (TME). Its underlying mechanism involves augmenting tumor-related immunity. TLS contributes to the activation of anti-tumor immune responses by promoting an inflamed tumor microenvironment, characterized by increased immune signatures including cytolytic activity, IFN-γ signaling, and MHC expression (16). Additionally, TLS is associated with a higher degree of immune cell infiltration within various tumor types, encompassing cytotoxic T lymphocytes, natural killer cells, and gamma delta T cells (22). Furthermore, the presence of mature dendritic cells, helper T cells, and B cells within TLS serves as a persistent source of stimulation for T cells linked to TLS (23).

It is worth noting that the presence of these immune cells also influences TLS activity. In a study on tumor-induced TLS in NSCLC patients, a higher density of Treg cells within TLS was associated with shorter patient survival, diminishing the favorable prognostic value of TLS (24). In another NSCLC study, the negative impact of high Treg cell density on patient survival was mitigated by a high density of B cells within TLS, with patients exhibiting a high B cell to low Treg cell ratio in TLS showing the best clinical outcomes (25). During the maturation of TLS, myeloid cells (such as mature dendritic cells), stromal cells (including follicular dendritic cells and follicular reticular cells), high endothelial venules, as well as B cells and T cells, all play indispensable roles (26). The functional differences of TLS are determined by the composition and proportion of its constituent cell types.

Interestingly, in a study on advanced-stage bladder cancer, CXCL13 was demonstrated as a surrogate marker for tumor TLS (27). As a chemoattractant, CXCL13 facilitates the formation of TLS and plays an important role in anti-tumor activity through the CXCL13/CXCR5 axis (28) and T follicular helper cells (29). However, further research is needed to explore its role in other solid tumors.

TLSs are ectopic lymphoid structures with a cellular composition similar to that of secondary lymphoid organs (SLOs), and therefore have comparable anti-tumor functions. However, TLSs also exhibit distinct characteristics that differentiate them from SLOs. Notably, unlike SLOs, TLSs are not encapsulated and are located within or around tumors, allowing them to exert a more direct and potent impact on the TME (26). This proximity may offer significant advantages in enhancing the effectiveness of ICI therapy.

TLS represents the continuous activation of the immune system during the anti-tumor immune process, thereby enhancing the body’s overall immune response to the tumor. TLS, while linked to improved anti-tumor responses post-ICI therapy, is also closely associated with the incidence of immune-related adverse events (irAEs). In clinical research, TLS has been observed in the tissues of patients experiencing acute interstitial nephritis (AIN), a severe form of irAE (30). Additionally, studies have shown that in aged, tumor-bearing mice, therapy targeting the programmed death receptor (PD)-1 can induce irAE-like symptoms and multiorgan dysfunctions characterized by TLS-like lymphocytic infiltration in affected organs, suggesting a correlation between TLS presence and irAE incidence in patients treated with immune checkpoint blockade (ICB) (31). This evidence underscores the importance for clinicians to actively monitor TLS density and be especially vigilant for irAEs in patients with high TLS levels, enhancing patient care by anticipating and managing potential adverse reactions.

In the studies we included, we sought to identify the similarities and differences of TLS across different tumor types. However, discrepancies in the detection methods of TLS pose significant challenges. We hope that future research will employ consistent histological markers for TLS identification.

Our findings showed that higher TLS density is linked to prolonged PFS. However, overall survival (OS) is also a common cancer survival index. OS is usually assessed alongside PFS to evaluate therapeutic efficacy in clinical research. In the currently available studies, high TLS density was consistently associated with longer OS (8, 10, 21).

Anti-PD1/PD-L1 antagonists are the most commonly used ICI treatments. However, in two studies on NSCLC and EC (12, 19), PD-L1 TPS/CPS did not show significant predictive value for ICI treatment efficacy. In contrast, our study found that TLS density is a significant predictor of ICI therapy effectiveness. This may be because TLS better reflects an individual’s immune status, which is crucial in determining response to ICI therapy. The ICI-insensitive state is essentially an immune condition independent of tumor type, highlighting the importance of individual immune characteristics (32). Compared to markers like PD-L1 TPS/CPS and tumor mutational burden (TMB), TLS provides a more personalized assessment of immune status (33). Despite this, no studies have directly compared the predictive value of PD-L1 TPS/CPS with that of TLS, leaving it uncertain which marker is more reliable for predicting ICI therapy efficacy. Further research is needed to clarify this issue.

Apart from acting as markers of therapeutic efficacy, TLSs present significant potential as direct targets for immunotherapy. Current research efforts are investigating ways to induce the formation of TLS through diverse approaches, such as employing STING agonists, inhibiting endothelial Notch signaling, and implementing systemic delivery of αCD40 (34–36). These exploratory studies are designed to synthesize and assess the resulting data, aiming to lay the groundwork for the creation of more dynamic and potent treatments. This forward-thinking strategy seeks to transform cancer management by more effectively leveraging the body’s immune system, offering a promising avenue for enhancing patient outcomes through immunologically centered interventions.

This study has several limitations. Primarily, our statistical analysis and validation efforts are centered on PD-L1, owing to the restricted use of other immune checkpoint inhibitors (ICIs) such as EGFR and HER2. This specificity suggests that our conclusions may not be universally applicable to treatments involving these other ICIs, advising a degree of caution in their broader application. Secondly, there is publication bias, but after statistical analysis, there is no effect on the stability of the results. Finally, the included studies and patients are limited, and our analyses are all based only on observational studies, potentially leaving some variables uncontrolled, highlighting the necessity for these results to be corroborated through large-scale, prospective studies dedicated to this area of research in the future.

## Conclusion

Our research demonstrates a significant correlation between TLS density and the efficacy of ICI therapy, underscoring the potential of TLS density as a valuable predictor for treatment outcomes. Given these findings, we advocate for clinicians to routinely assess TLS density in cancer patients. Those identified with high TLS density may particularly benefit from ICI treatment. This approach could enable more personalized and effective immunotherapy strategies, potentially improving patient responses and outcomes.

## Data Availability

The original contributions presented in the study are included in the article/**Supplementary Material**. Further inquiries can be directed to the corresponding author.
